# Significance of the multiomics approach to elucidate disease mechanisms in humans

**DOI:** 10.1186/s41232-022-00227-5

**Published:** 2022-11-04

**Authors:** Keishi Fujio

**Affiliations:** Department of Allergy and Rheumatology, Graduate School of Medicine, The University of Tokyo, Tokyo, Japan

The basis of many human diseases is inflammation of various organs. Inflammation of the blood vessels induces cerebrovascular disease, and chronic inflammation of organs is a breeding ground for carcinogenesis. Inflammation in organs such as the lungs, joints, and muscles forms organ-specific autoimmune diseases such as interstitial pneumonia, arthritis, and idiopathic inflammatory myopathy. Therefore, clarification of the mechanism of inflammation would lead to the selection and development of effective therapies. However, inflammation involves various types of immune cells and parenchymal or stromal cells, as well as many genes that function on these cells. Therefore, elucidating the mechanisms of such inflammation is a daunting task.

Knockout mice are one of the most robust research approaches, and this experimental system has revealed that modification of a single gene can lead to specific inflammatory diseases. It was also clearly demonstrated that even though the pathologic picture of inflammation may appear similar, the causal genes can differ substantially. However, the problem is that the systems of humans, who live 80 years, and mice, which live only one year, are very different. The findings obtained in mice are not necessarily applicable to humans. Therefore, to understand human inflammation, it is important not only to elucidate the phenotype of gene-deficient mice, but also to obtain more detailed information on the status of human inflammation. In particular, genome analysis, including genome-wide association study, is a very powerful tool to identify susceptibility genes and susceptibility polymorphisms that cause diseases. Furthermore, recent technological advances, including the development of next-generation sequencers, have made it possible to obtain multiomics information, including genomic information. Multiomics analysis is expected to elucidate the pathogenesis of various inflammatory diseases in humans.

In this thematic series review, we invited the leading researchers in this research field. Dr. Shirai from Osaka University reviewed the useful data resources for connecting omics layers and describes how they are combined into a cohesive analysis. This review clarifies how the combination of transcriptome, single cell analysis, proteome, metabolome, and epigenome empowers the information from the individual omics.

Dr. Nagasawa from The University of Tokyo reviewed the recent progress of single-cell cancer analysis in the context of drug resistance. In addition, spatial transcriptome (ST) analysis is described in an easy-to-understand manner as an analysis method to supplement the spatial position information lost in single cell analysis.

Dr. Yamashita of Tokyo Medical and Dental University provided an overview of primary immunodeficiency (PID), a disease derived from a single gene mutation in humans. Recent advances in genetic analysis, including whole-exome analysis, whole-genome analysis, and RNA-seq enabled the identification of the disease-causing gene mutation in humans. This approach is very powerful in accomplishing both the elucidation of the cause of the disease and its diagnosis.

Dr. Tsuchiya from The University of Tokyo reviewed the epigenetic abnormalities in synovial fibroblast in rheumatoid arthritis. By focusing on epigenomic modifications and gene expression associated with genetic risk for RA, the identification of important immune pathways leading to patient stratification will be realized.

Here, we would like to express sincere appreciation to the distinguished researchers who contributed to this special issue. We really hope that these prominent articles will provide novel insights to the researchers in the field of inflammation and regeneration.

